# A Comparative Neuroanatomical Study of the Red Nucleus of the Cat, Macaque and Human

**DOI:** 10.1371/journal.pone.0006623

**Published:** 2009-08-13

**Authors:** Satoru Onodera, T. Philip Hicks

**Affiliations:** 1 Department of Anatomy, School of Medicine, Iwate Medical University, Morioka, Japan; 2 Department of Biology, Faculty of Science and Environmental Studies, Lakehead University, Thunder Bay, Ontario, Canada; Duke Unviersity, United States of America

## Abstract

**Background:**

The human red nucleus (Nr) is comparatively less well-studied than that of cats or monkeys. Given the functional importance of reticular and midbrain structures in control of movement and locomotion as well as from an evolutionary perspective, we investigated the nature and extent of any differences in Nr projections to the olivary complex in quadrupedal and bipedal species. Using neuroanatomical tract-tracing techniques we developed a “neural sheet” hypothesis allowing us to propose how rubro-olivary relations differ among the three species.

**Methods and Findings:**

Wheat germ agglutinin-horseradish peroxidase staining supports findings that the cat's nucleus accessories medialis of Bechtrew (NB) projects mainly to the lateral bend of the principal olive. We clarified boundaries among nucleus of Darkschewitsch (ND), NB and parvicellular red nucleus (pNr) of the cat's neural sheet. The macaque's ND-medial accessory olivary projection is rostro-caudally organized and the dorsomedial and ventrolateral parts of the macaque's pNr may project to the principal olive's rostral and caudal dorsal lamella; in cat it projects as well to pNr. Myelin- and Nissl-stained sections show that a well-developed dorsomedial part of the human Nr consists of densely packed cells, deriving small myelinated fibers that continue into the medial central tegmental tract.

**Conclusions:**

Based on these findings we suggest there are distinct bipedal-quadrupedal differences for Nr projections to the olivary complex. We propose the Nr of cats and monkeys comprise the ND, NB and pNr in a zonal sheet-like structure, retaining clear nuclear boundaries and an isolated, well-developed mNr. The human NB may be distinguished from its more specialised ND (ND lies alongside a well-developed pNr) in the human central gray. Phylogenetically, the NB may have been translocated into a roll-shaped Nr in the reticular formation, the dorsomedial portion of which might correspond to the cat's and monkey's NB.

## Introduction

The structure and function of the mesodiencephalic region and possible homologies among experimental species used in neuroanatomical and electrophysiological studies such as rat, cat and monkey has long been problematic. However the important role of many of the midbrain nuclei in locomotion especially, but also in many aspects of other forms of motor control, make a comparison of connectivity relationships involving this region between bipeds and quadrupeds of considerable interest. Such knowledge may lead for example to new insights into evolutionary processes which could have resulted in the advent of bipedalism. They also may cast new light on how fine-movement control likely emerged from more primitive forms of template-based motor functions. The aim of the present study was to identify area(s) corresponding to the human nucleus accessories medialis of Becheterew (NB) and its descending tract through comparisons of data from the cat, macaque and human, and to propose a new model for the human red nucleus.

In humans, the red nucleus consists of the parvicellular red nucleus (pNr) and it derives the central tegmental tract (CTT), whereas the cat's red nucleus contains the magnocellular red nucleus (mNr), the origin of the rubrospinal tract. In a pioneering study of this region in cats, Ogawa (1939) proposed that the cat's pNr corresponded to a nuclear complex consisting of the nucleus of Darkschewitsch (ND), the interstitial nucleus of Cajal (Nint) and the nucleus of the fields of Forel, forming the medial tegmental tract (MTT) [1]. Mannen (1988) published a work entitled “A dendro-cyto-myeloarchitectonic atlas of the cat's brain”; his work was based on material stained by Golgi-, Weigert- and Nissl-prepared serial sections [2]. Individual Golgi-impregnated neurons (printed in eight published colours) clearly were identifiable throughout the entire brain. This excellently detailed atlas showed the cat's mNr as being classified as “closed nuclei” in contrast to “open nuclei”, since the extranuclear dendrites of its cells seldom were found in the neighboring reticular formation. In these normally stained sections, the poorly-developed feline pNr could not be clearly demarcated from among the diffusely distributed neurons of the reticular formation. Of course all anatomical studies, whether using tract-tracing with WGA-HRP or cytological in nature (e.g., Golgi-, Weigert- or Nissl-staining) particularly those done in earlier decades, will be limited by technical and methodological issues that make it difficult to draw firm conclusions about nuclear boundaries or patterns of connectivity.

However one of us was able to demonstrate the feline pNr as consisting of a long cellular zone continuing from the ND to the rostral part of the mNr through the use of an HRP-tracing method (Onodera, 1984 [3]). After HRP deposition into the central core of the cat's inferior olive, almost all the cells of both the ND and NB, located in the ventral central gray. A number of labelled small cells also were found in both the dorsomedial (dm)- and ventrolateral (vl)-pNr of the reticular formation. The cat possesses a continuous zonal neural sheet consisting of the ND, NB and the poorly-developed dm- and vl-pNr and it is readily seen that this mNr is well-developed for quadrupedalism (Onodera, 1984 [3]).

mNr neurons in quadrupeds (e.g., cat: Drew et al., 1996 [4]) exhibit a significant increase in discharge frequency during of either fore- or hindlimbs movement when the animals cross obstacles, similar to what is seen in pyramidal tract neurons. These neurons are controlled by a network resembling a continuous zonal neural sheet, i.e., the mesodiencephalo-olivo-cerebellar system. Such animals execute voluntary gait modification of fore- and hindlimbs using visual and other sensory cues [5]. Pyramidal tract neurons contribute to fine, precise control of distal limb movements both by increases and decreases in the level of spinal interneuron activity. By contrast, mNr neurons may discharge more in relation to intralimb coordination as well as interlimb coordination [5]. mNr neurons of rats and monkeys, species that possess a manipulable hand, are provided with a “hand preshape” through their extension of digits via goal-directed limb movements, such as reaching to grasp [6], [7], [8], [9]. Pyramidal tract neurons which are controlled by a continuous zonal neural sheet-olivo-cerebellar circuit, superimpose motor control for individual finger movements on grouped-finger extension, such action which is governed by rubrospinal tract neuron activity.

In the bipedal human, the mNr is rudimentary. From an evolutionary perspective, human pyramidal tract neurons controlled by the rubro-olivo-cerebellar circuit would have subsumed the function of the framework governing limb movements that, until then, had been provided by the rubrospinal tract neurons, thereby introducing stability and accuracy of skilled movements of all muscle groups required for locomotion, enabling the advent of bipedalism. Although the human ND is identified as a separate body part from the well-developed red nucleus, demarcated by fiber bundles consisting of the superior cerebellar peduncle, CTT, MTT and the medial longitudinal fasciculus (MLF), the human NB could not be identified in the ventral central grey, as is seen for the cat's NB.

## Materials and Methods

Two adult female and male cats and three female macaques (*Macaca fuscata*) were employed as subjects in the present study. All experimental protocols were performed in accordance with the Animal Experimental Guidelines of Iwate Medical University and the Canadian Council of Animal Care, and the ethics protocol was approved by the Animal Users Subcommittee of Iwate Medical University. Procedures were designed to minimize animal suffering and reduce the number used. Human brains were obtained via informed donation for medical education and research of Iwate Medical University with the corresponding written consents given by donors and their families who agreed with policies relating to good–will cadaver donation. Human brain slice preparation approved by the Ethical Committee of Iwate Medical University is used annually in neuroanatomical courses at Iwate Medical University; cadaver identity data are anonymous. All experiments were performed between 1996 and 1998.

### Animal Preparation

Two cats in Case 1 (female, 2.8 kg) and Case 2 (male, 3.0 kg) and two macaques (*Macaca fuscata*) in Case 3 (female, 9.0 kg), and Case 4 (female,10.0 kg) were used for the demonstration of mesodiencephalic nuclei consisting of olivary projecting neurons. One macaque in Case 5 (female, 10.5 kg) was used for the demonstration of the cortico-mesodiencephalic projection.

After induction of sedation by an intramuscular injection of ketamine hydrochloride (10 mg/kg body weight), animals were anesthetized deeply by intraperitoneal injections of sodium pentobarbital (30 mg/kg body weight). Then, atropine sulphate (60 µg/kg body weight) was injected intramuscularly. To gain sterotaxic access to the inferior olivary complex (Case 1–4), part of the occipital bone lying over the occipital cortex and cerebellum was removed. For demonstration of mesodiencephalic nuclei consisting of olivary projecting neurons, these animals received multiple stereotaxic injections (0.2 µl/one injection for the cat, 1.0 µl/one injection for the macaque) of 2% WGA-HRP (Toyobo, Tokyo, Japan) solution as retrograde tracer via a 1-µl Hamilton microsyringe into the entirety of the olivary complex (two cats, Cases 1 and 2: 0.2 µl×2 and 0.2 µl×4) or with the use of a 5-µl Hamilton microsyringe into its subnuclei (two macaques, Cases 3 and 4: 1.0 µl×6).

A large craniotomy was made to expose the appropriate region of frontal cortex of one macaque (Case 5). For demonstration of the cortico-mesodiencephalic projection, this animal received, under direct visual control, multiple injections (23 sites) of 10.6 µl 2% WGA-HRP solution as anterograde tracer using a 1-µl Hamilton microsyringe into the premotor area and frontal eye field (FEF).

Animals were permitted to survive for 2 days after the injections, and were overdose-anesthetized prior to transcardial perfusion with 500 ml of warmed (37°C) physiological saline followed by 1,500 ml (for cats) or 2,500 ml (for macaques) of fixative containing 1% paraformaldehyde and 2.5% glutaraldehyde in 0.1 M phosphate buffer (PB), pH 7.4 and then by 1,000 ml PB, pH 7.4. Whole brains were stored overnight at 4°C in 0.1 M PB containing 30% sucrose. Coronal sections of 50 µm thickness were cut with a vibratome or a freezing microtome and collected in 0.1 M PB. Sections were processed for the histochemical demonstration of HRP with the tetramethylbenzidine method [10] and counterstained with neutral red. The distribution of labeled axons and somata were photographed and drawn through the use of a photomicroscope (Olympus) with a drawing tube attached.

### Human Brain Preparation

After death and within 24 hrs postmortem, cadavers were perfused through the femoral artery with 10,000 ml of a 10% formalin solution. Brains were removed immediately from the skulls and immersed in the same fixative for several months. Well-fixed adult brain was selected and washed in running water. Macro- and microscopical examination of this brain did not reveal any lesions. The diencephalon and brainstem were cut into several blocks. These blocks were cut by a freezing microtome into 50 µm-thick serial coronal sections. Each section was picked up with a chrome alum gelatin-coated slide. Every twelfth section was stained with 1% cresyl violet. Other serial sections were stained with myelin stain using Luxol fast blue. The distributions of Nissl stained cells were plotted using a drawing tube.

### Drawing of 3D Image

Frontal, dorsal and lateral views of the structures of the mesodiencephalic nuclei of the cat, macaque and human were drawn from the cat's and macaque's WGA-HRP labelled serial sections for cat, macaque and human (herein illustrated sequentially) using Nissl stained serial sections. Three-dimensional images were drawn freehand according to these frontal, dorsal and lateral views.

## Results

### Structure of the Cat's Red Nucleus

#### Olivary injection sites (Fig. 1)

**Figure 1:**
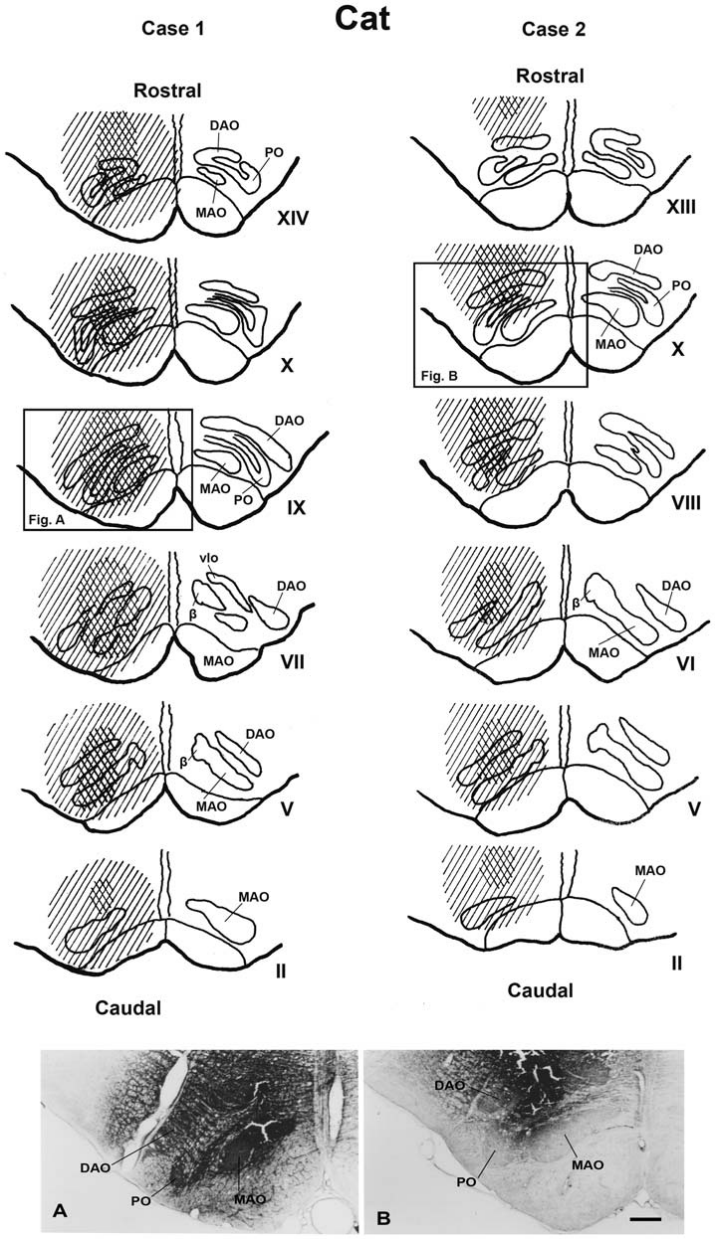
WGA-HRP injection sites in cat's inferior olivary complex (case 1 and 2). The upper two columns comprise a scale drawing of the distribution of injected WGA-HRP solution in the cat's inferior olivary complex in case 1 and 2. The Roman numerals (II-XIV) represent levels of the inferior olivary complex from caudal to rostral (Brodal, 1940 [60]). The crossed stripes indicate a heavily stained area. Lower photomicrographs show the maximal extent of injected wheat germ agglutinin-horseradish peroxidase (WGA-HRP) at the olivary level (Fig. 1) in case 1 (A) and case 2 (B). DAO–dorsal accessory olive, MAO–medial accessory olive, PO–principal olive, vlpNr–ventorlateral part of parvicellular red nucleus, β–nucleus β. Scale bar = 500 µm in B (also applies to A).

In case 1, where there were four, 0.2-µl injections performed and the tissue processed 44 hrs later, the injection site was found to be centered into the cat's olivary complex (see Fig. 1A). All olivary subnuclei were heavily stained throughout the rostro-caudal direction with additional partial involvement of the surrounding reticular formation.

In case 2, where there were two, 0.2-µl injections performed and the tissue processed 43 hrs later, the injection site was found also to be centered into the cat's olivary complex (see Fig. 1B), however this injection was dorsally shifted compared with case 1. Therefore this tracer strongly stained the dorsal accessory olive (DAO), the dorsal lamella (dl) of the principal olive (PO) and the caudal part of the medial accessory olive (MAO), and extended as well to the medullary reticular formation, although the lateral bend of PO and the ventral-most part of rostral MAO were free from staining.

#### Distribution of labeled cells in the mesodiencephalic area (Figs. 2 and 3)

**Figure 2:**
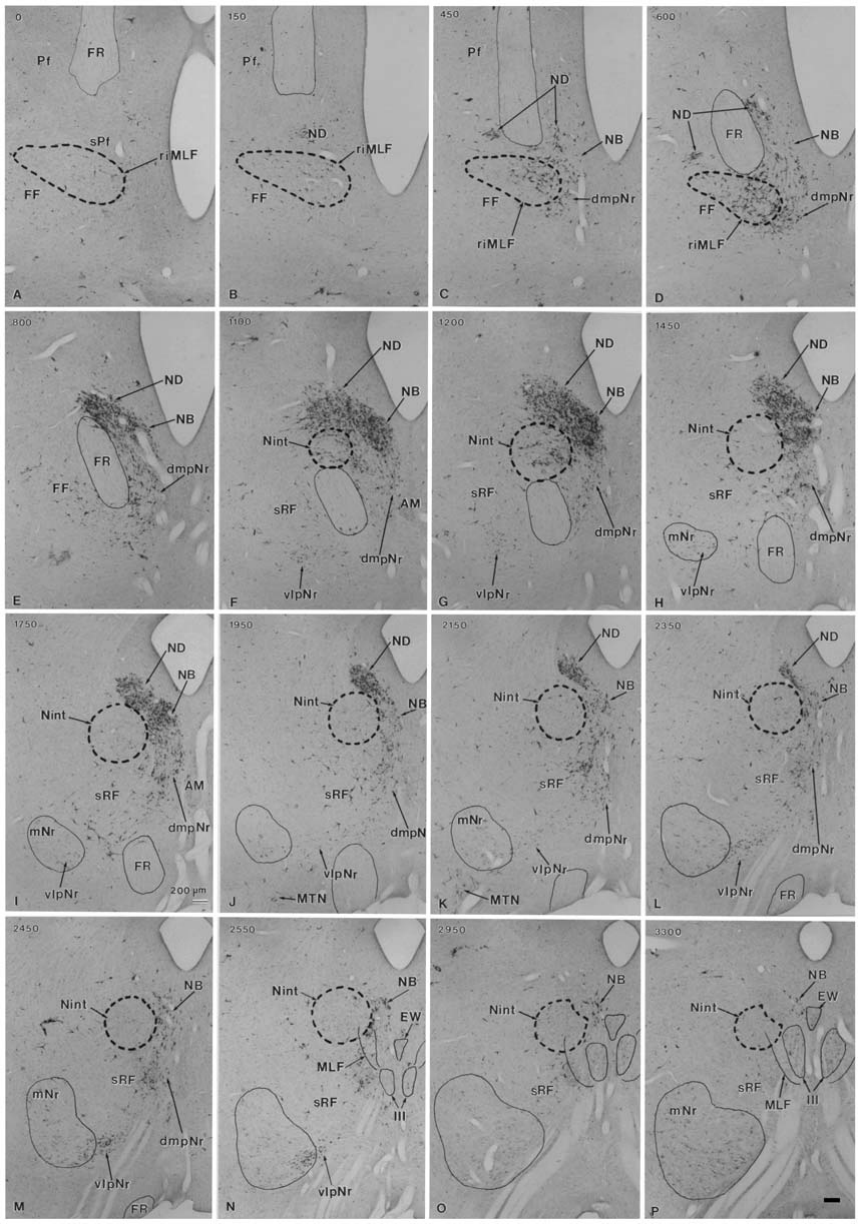
Distribution of WGA-HRP-labelled cells in mesodiencephalic structures (case 1). Successive serial sections are rostrally arranged, i.e., section A is the most rostral. The number in the left corner of each photomicrograph is the rostrocaudal distance (in micrometers) from section A. AM–anteromedian nucleus, dmpNr–dorsomedial part of parvicellular red nucleus, EW–Edinger-Westphal nucleus, FF–Field of Forel, FR–fasciculus retroflexus, MLF–medial longitudinal fasciculus, mNr–magnocellular red nucleus, MTN–medial terminal nucleus, NB–nucleus accessorius medialis of Bechterew, ND–nucleus of Darkschewitsch, Nint–interstitial nucleus of Cajal, Pf–parafascicular nucleus, riMLF–rostral interstitial nucleus of MLF, sPf–subparafascicular nucleus, sRF–suprarubral reticular formation, vlpNr–ventorlateral part of parvicellular red nucleus, III–oculomotor nucleus. Scale bar = 200 µm in P (also applies to A–O).

The heavily labelled ND (sections B–L) and NB (sections C–P) were found ipsilaterally in the ventral central gray, while the labelled dmpNr (sections C–M) and vlpNr (sections F–N) were located ipsilaterally in the reticular formation. All sections in this paragraph refer to Fig. 2. At the rostral level of the ND and NB (sections C–E), these nuclei fuse with each other and at this level the border between these structures is unclear. A narrowing of the nuclear zones is observed medially (sections F–J). The separation between ND and NB occurs at caudal levels (sections K and L). One of the causes of the separation between ND and NB might be the increase that occurred in number of descending fibers emerging from these nuclei and the consequent constriction of ND. The distal tip of ND did not extend to the level of the oculomotor nucleus (section M). The distal tip of NB exits dorsally to the rostral part of the oculomotor nucleus (sections N–P). The fibers of the fasciculus retroflexus (FR) penetrate the lateral side of the rostral tip of the ND (sections C and D), pass through the lateral side of the rostral ND and NB (sections C–E), between the rostral interstitial nucleus of MLF and Nint (sections D–F) and penetrate between dmpNr and vlpNr (sections F–H). The lateral part of the vlpNr overlaps with the rostral part of the mNr (sections H–N).

**Figure 3:**
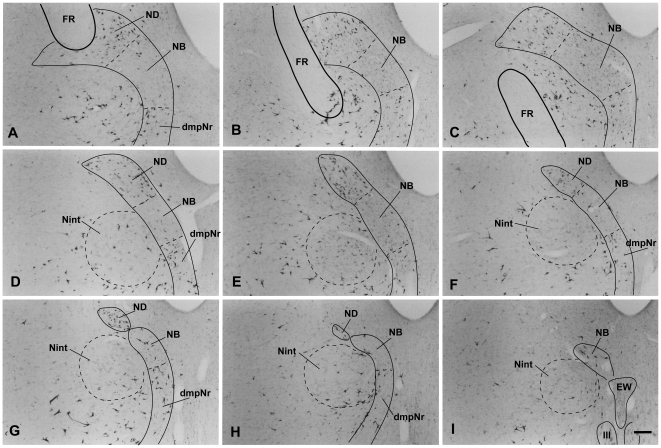
Distribution of WGA-HRP-labelled cells in mesodiencephalic structures (case 2). Successive serial sections are rostrally arranged, i.e., section A is the most rostral. dmpNr–dorsomedial part of parvicellular red nucleus, EW–Edinger-Westphal nucleus, FR–fasciculus retroflexus, NB–nucleus accessorius medialis of Bechterew, ND–nucleus of Darkschewitsch, Nint–interstitial nucleus of Cajal, III–oculomotor nucleus. Scale bar = 200 µm in I (also applies to A–H).

In case 2, the pattern of the distribution of labeled cells was fundamentally similar to case 1. However, due to the fact that the number of labeled neurons of the NB were prominently reduced compared with case 1, the border of the NB was more readily definable along its rostrocaudal axis (see Fig. 3).

Because the major difference between cases 1 and 2 concerning the involvement of the lateral bend of the PO, the results indicate that NB projects to this olivary region.

### Structure of the Macaque's Red Nucleus

#### Olivary injection sites (Fig. 4)

Case 3 comprised 6 injections of 1.0-µl, a 45-hr wait for tissue preparation and resulted in an injection site that encompassed the rostral half of the olivary complex. The tracer was restricted to the rostral parts of MAO, DAO and PO, with additional partial involvement of the surrounding reticular formation. Therefore the rostral (XII–VIII level) part of dl of PO was heavily stained, but the caudal (VII–V level) part of dl of PO was stain-free.

**Figure 4:**
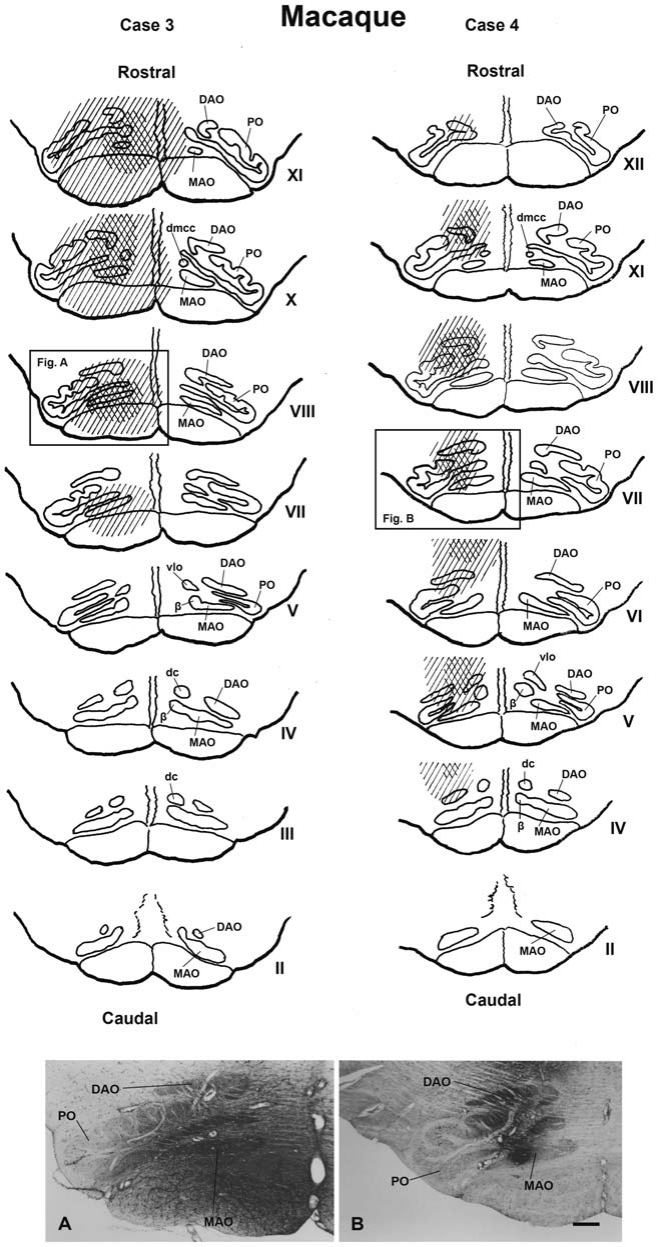
WGA-HRP injection sites in macaque's inferior olivary complex (case 3 and 4). The upper two columns comprise a scale drawing of the distribution of injected WGA-HRP solution in the macaque's inferior olivary complex in case 3 and 4. The Roman numerals (II–XII) represent levels of the inferior olivary complex from caudal to rostral (Brodal and Brodal, 1981 [61]). The crossed stripes indicate a heavily stained area. Lower photomicrographs show the maximal extent of injected WGA-HRP at the olivary level in case 3 (Fig. A) and case 4 (Fig. B). DAO–dorsal accessory olive, dc–dorsal cap, dmcc–dorsomedial cell column, MAO–medial accessory olive, PO–principal olive, vlo–ventrolateral outgrowth, β–nucleus β. Scale bar = 500 µm in B (also applies to A).

Case 4 also comprised 6 injections of 1.0-µl and a 45-hr wait for tissue preparation. It encompassed the middle part of the olivary complex. The rostral (XII-XI level) part of dl-PO and most of the MAO were stain-free. The caudal (VIII–V) part of dl of PO and a restricted spot in the middle of MAO were heavily stained, with additional partial involvement of the surrounding reticular formation.

#### Distribution of labeled cells in the mesodiencephalic area (Figs. 5,6,7)

**Figure 5:**
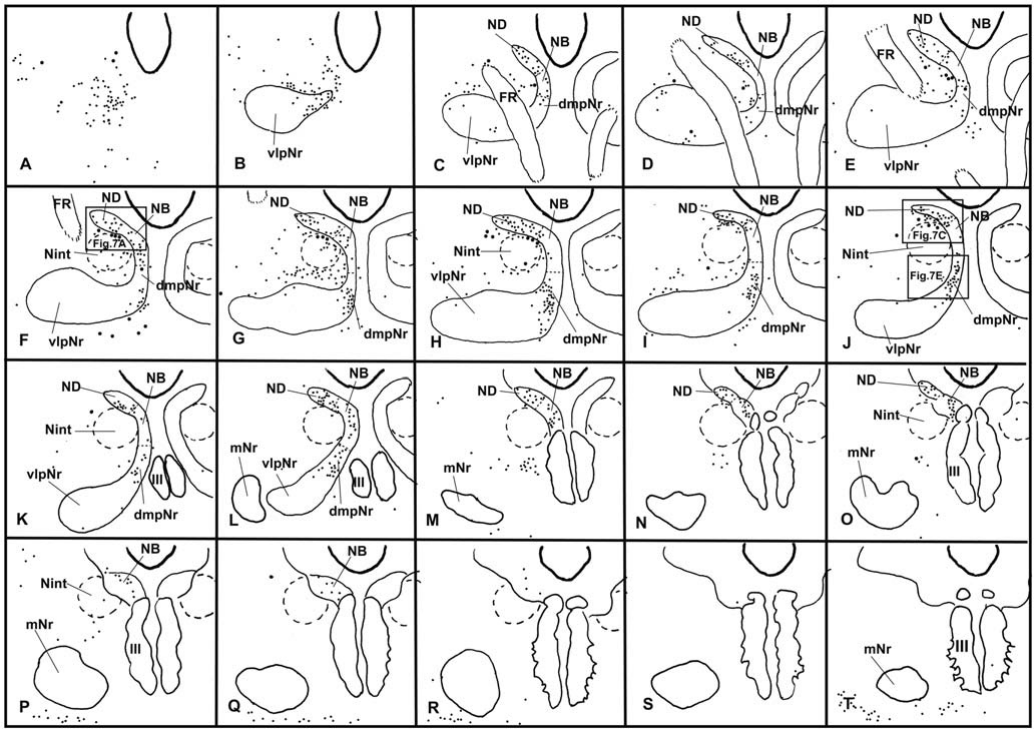
Distribution of WGA-HRP-labelled cells in mesodiencephalic structures (case 3). Drawings are arranged rostrocaudally; section A is the most rostral. One dot represents one labelled cell. Large dot shows a large labelled cell. dmpNr–dorsomedial part of parvicellular red nucleus, FR–fasciculus retroflexus, mNr–magnocellular red nucleus, NB–nucleus accessorius medialis of Bechterew, ND–nucleus of Darkschewitsch, Nint–interstitial nucleus of Cajal, vlpNr–ventorlateral part of parvicellular red nucleus, III–oculomotor nucleus.

**Figure 6:**
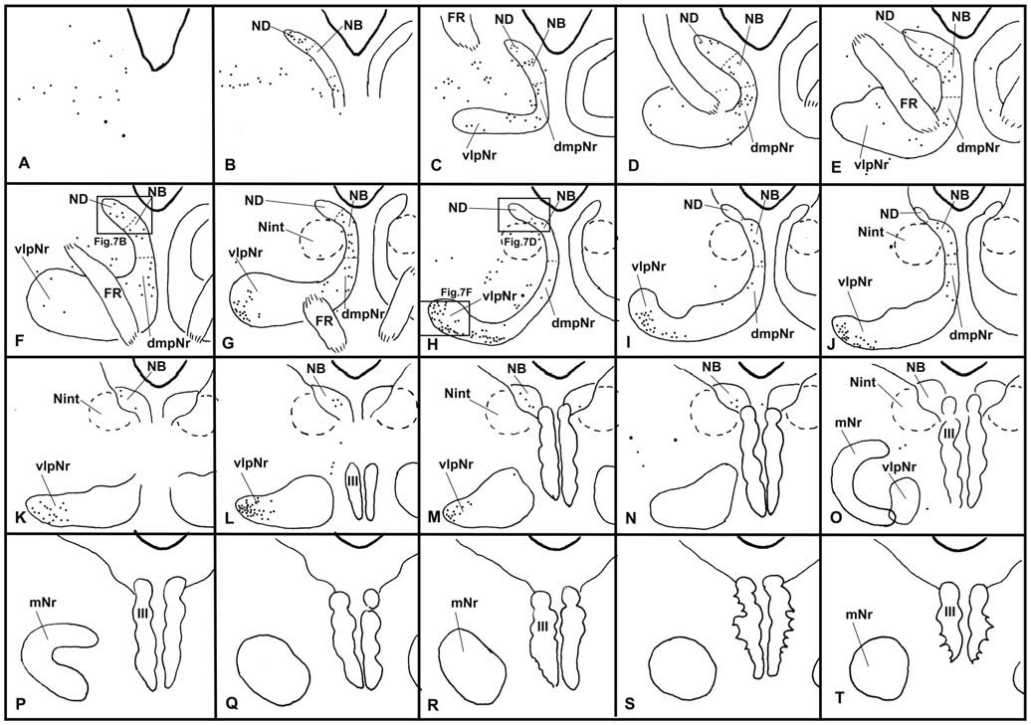
Distribution of WGA-HRP-labelled cells in mesodiencephalic structures (case 4). Drawings are arranged rostrocaudally; section A is the most rostral. One dot represents one labelled cell. Large dot shows a large labelled cell. dmpNr–dorsomedial part of parvicellular red nucleus, FR–fasciculus retroflexus, mNr–magnocellular red nucleus, NB–nucleus accessorius medialis of Bechterew, ND–nucleus of Darkschewitsch, Nint–interstitial nucleus of Cajal, vlpNr–ventorlateral part of parvicellular red nucleus, III–oculomotor nucleus.

**Figure 7:**
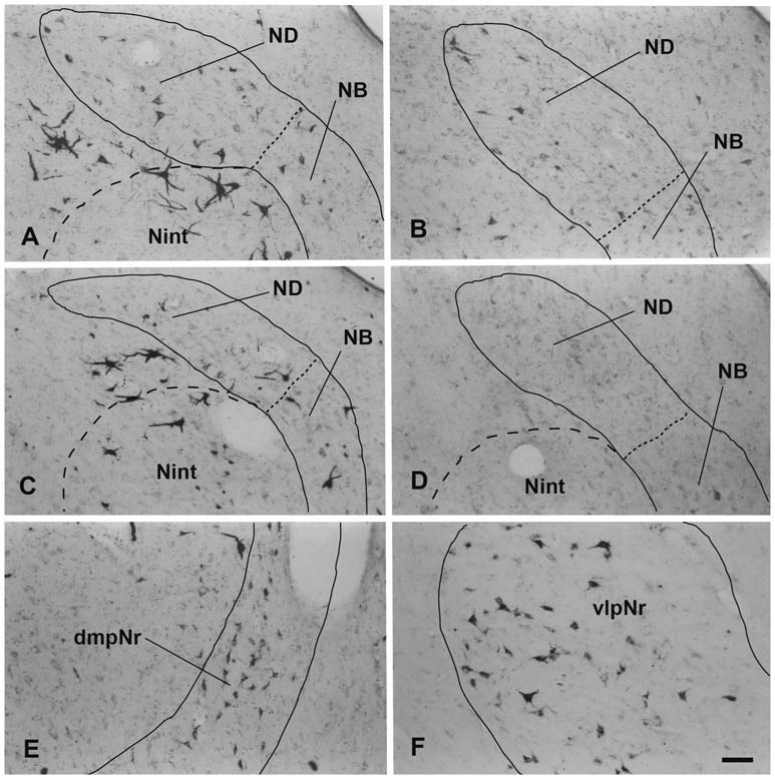
Distribution of WGA-HRP-labelled cells in mesodiencephalic structures (case 3 and 4). A (case 3) and B (case 4): rostral part of ND, C (case 3) and D (case 4): caudal part of ND. E (case 3): dmpNr. F (case 4): vlpNr. dmpNr–dorsomedial part of parvicellular red nucleus, NB–nucleus accessorius medialis of Bechterew, ND–nucleus of Darkschewitsch, vlpNr–ventorlateral part of parvicellular red nucleus. Scale bar = 100 µm in F (also applies to A–E).

Labeled neurons in case 3 (Fig. 5) were observed ipsilaterally throughout the extent of the ND (Figs. 5, 7A and C), but some labeled cells were seen in the NB. Although many labeled cells were observed in the dmpNr (Figs. 5 and 7E), no labeled cells were observed in the well-developed vlpNr. No labeled cells from case 4 (Fig. 6) were found in the caudal half of ND (Figs. 6 and 7D), whereas some labeled cells were seen ipsilaterally in the dmpNr, NB and the rostral half of ND (Figs. 6 and 7B). Many labeled neurons were observed ipsilaterally in a restricted area of the well-developed vlpNr (Figs. 6 and 7F).

Owing to the major difference between cases 3 and 4 concerning the rostral part of both rostral MAO and dl of PO, the rostral and caudal parts of the ND can be said to project to the caudal and rostral parts of the rostral MAO, respectively, and the dmpNr and vlpNr project to the rostral and caudal part of the dl of PO, respectively.

### Fronto-Rubral Projection of the Macaque (Figs. 8 and 9)

**Figure 8:**
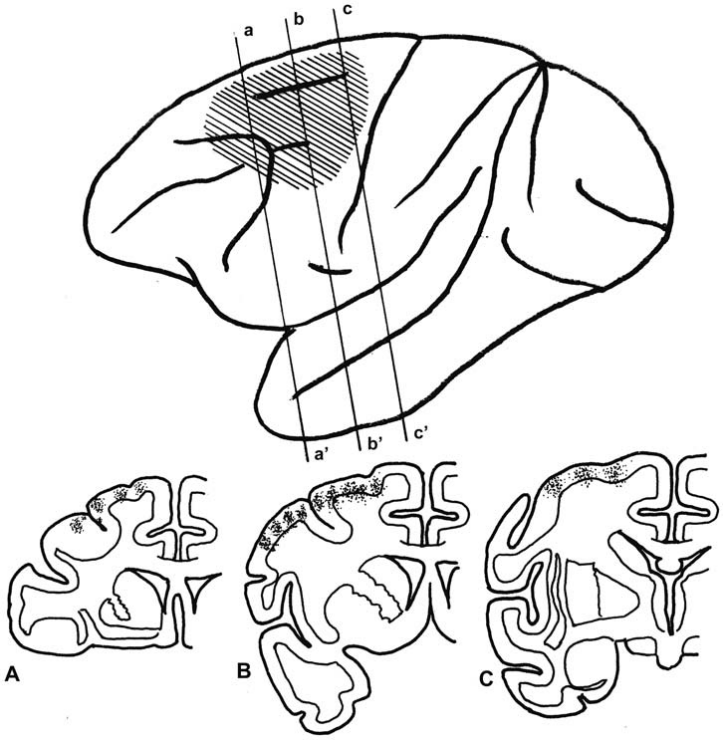
WGA-HRP injected sites in macaque's cerebral cortex (case 5). Upper drawing shows the expansion of the injected WGA-HRP solution indicated by oblique lines in a lateral view of the macaque's cerebral cortex. Lower drawing shows multiple injection sites (small dots) in the frontal section. A, B and C indicates a-a', b-b' and c-c' in upper drawing, respectively.

**Figure 9:**
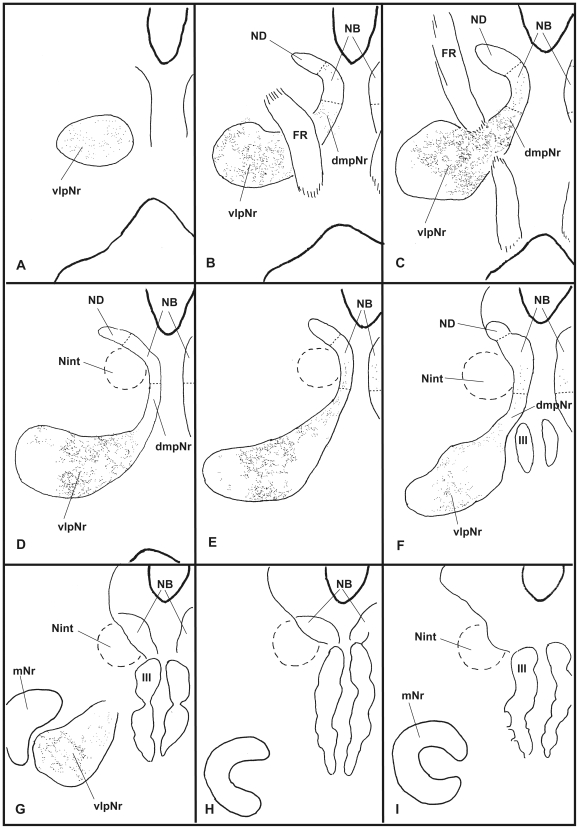
Distribution of WGA-HRP-labelled terminals in macaque's mesodiencephalic structures (case 5). Labelled terminals were indicated by small dots. dmpNr–dorsomedial part of parvicellular red nucleus, FR–fasciculus retroflexus, mNr–magnocellular red nucleus, NB–nucleus accessorius medialis of Bechterew, ND–nucleus of Darkschewitsch, Nint–interstitial nucleus of Cajal, vlpNr–ventorlateral part of parvicellular red nucleus, III–oculomotor nucleus.

Multiple injections of 10.6-µl WGA-HRP solution into 23 sites were performed into premotor and FEF areas, but sparing F5 (see Matelli et al., 1985 [11]) and the leg-foot region of the premotor area in the macaque (Fig. 8) for case 5, and after a 41-hr wait provided before tissue preparation. In the rostral diencephalons, labeled descending fibers appeared gathered in the medial part of the crus cerebri and scattered labeled descending fibers were observed ipsilaterally in the subthalmic nucleus, the zona incerta and adjacent thalamic nuclei. Many labeled terminal arborizations were seen ipsilaterally in the dmpNr and the medial part of the vlpNr, and some labeled terminal arborizations were observed in the NB, but few were observed in the ND, the Nint, the lateral part of the vlpNr or the mNr (Fig. 9). In the contralateral side a few labeled terminal arborizations were observed only in the NB (Fig. 9E and F). The more caudo-lateral parts of vlpNr were penetrated by larger fiber bundles of the superior cerebellar peduncle, judging from the size of the unlabelled area (compare Fig. 9C and G).

This case demonstrates that in the macaque, dmpNr and NB are targeted by the middle premotor and FEF regions, however projection to the ND has yet to be definitively shown. Owing to the unlabeled area, the leg-foot region of the premotor area can be said to project to the lateral part of the vlpNr, and F5 to the NB.

### Myelo- and Cytoarchitecture of the Human Red Nucleus (Figs. 10,11,12)

**Figure 10:**
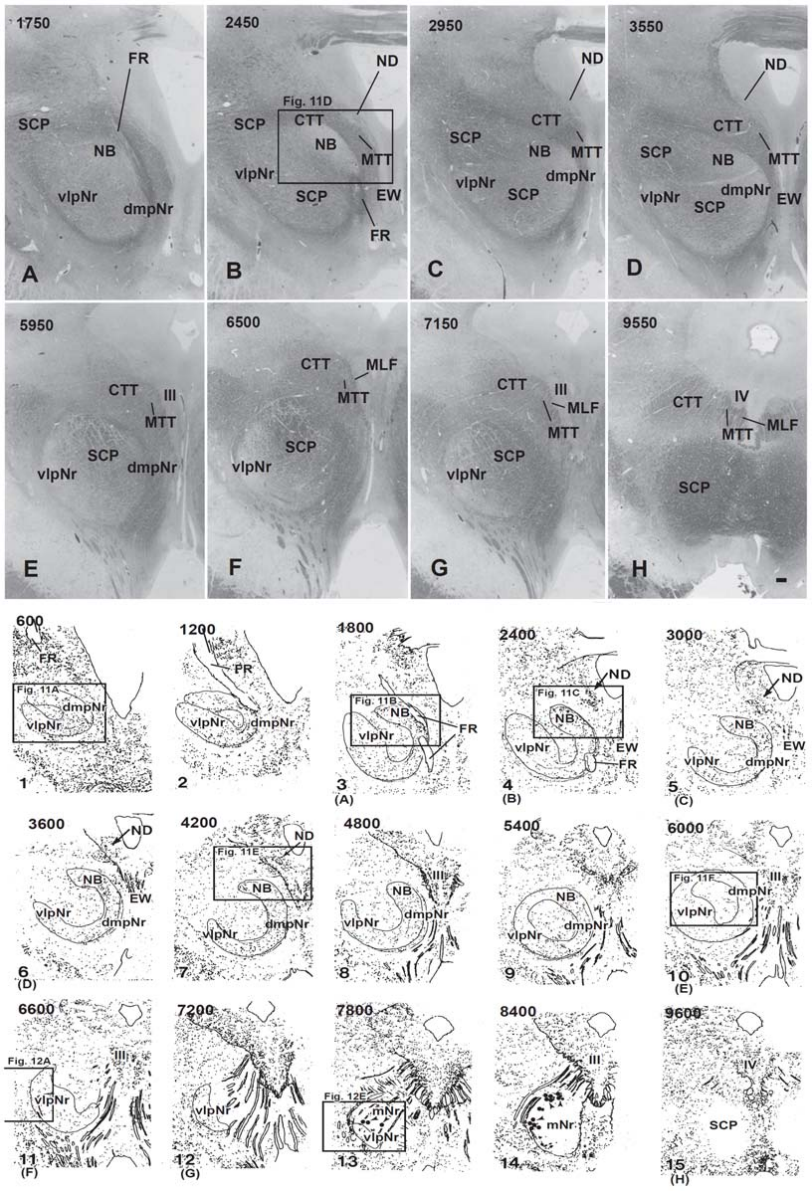
Human red nucleus and its surrounding structures. Upper photomicrographs (A-H) showing distribution of myelinated fiber bundles stained by myelin stain in the human mesodiencephalic structure of successive serial sections. Section A is the most rostral. The human NB is the dorsomedial cell-rich area of the red nucleus. Scale bar = 500 µm in H (also applies to A–G). Lower scale drawing showing the distribution of Nissl-stained cells found in the human mesodiencephalic structure as indicated by dots in the drawings of 15 successive serial sections. Section 1 is the most rostral. Sections 13 and 14 show giant mNr cells as large dots indicated by arrowheads. The number in the left corner of each photomicrograph and drawing is the rostrocaudal distance (in micrometers) from the rostral tip of the red nucleus. (A) – (H) correspond to Figs. 11A–H. CTT–central tegmental tract, dmpNr–dorsomedial part of parvicellular red nucleus, EW–Edinger-Westphal nucleus, FR–fasciculus retroflexus, MLF–medial longitudinal fasciculus, mNr–magnocellular red nucleus, MTT–medial tegmental tract, NB–nucleus accessorius medialis of Bechterew, ND–nucleus of Darkschewitsch, SCP–superior cerebellar peduncle, vlpNr–ventorlateral part of parvicellular red nucleus, III–oculomotor nucleus, IV–trochlear nucleus.

**Figure 11:**
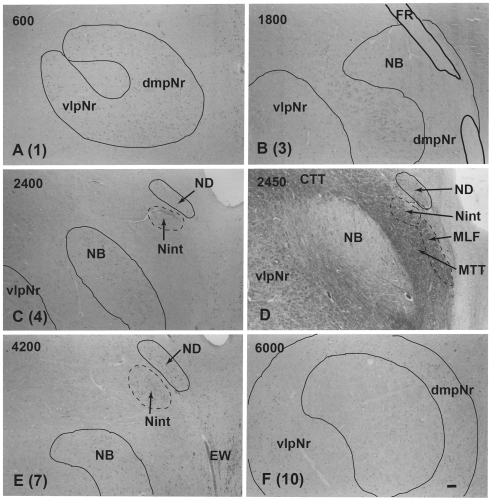
Human parvicellular red nucleus. Photomicrographs showing the distribution of Nissl-stained pNr cells in A(1), B(3), C(4), E(7), and F(10) which correspond to sections 1, 3, 4, 7 and 10 of Fig. 10 and the distribution of myelinated fiber bundles stained by myelin stain in D which corresponds to section Fig. 10B. The number in the left corner of each photomicrograph is the rostrocaudal distance (in micrometers) from the rostral tip of the red nucleus. CTT–central tegmental tract, dmpNr–dorsomedial part of parvicellular red nucleus, EW–Edinger-Westphal nucleus, FR–fasciculus retroflexus, MLF–medial longitudinal fasciculus, MTT–medial tegmental tract, NB–nucleus accessorius medialis of Bechterew, ND–nucleus of Darkschewitsch, Nint–interstitial nucleus of Cajal, vlpNr–ventorlateral part of parvicellular red nucleus. Scale bar = 200 µm in F (also applies to A–E).

**Figure 12:**
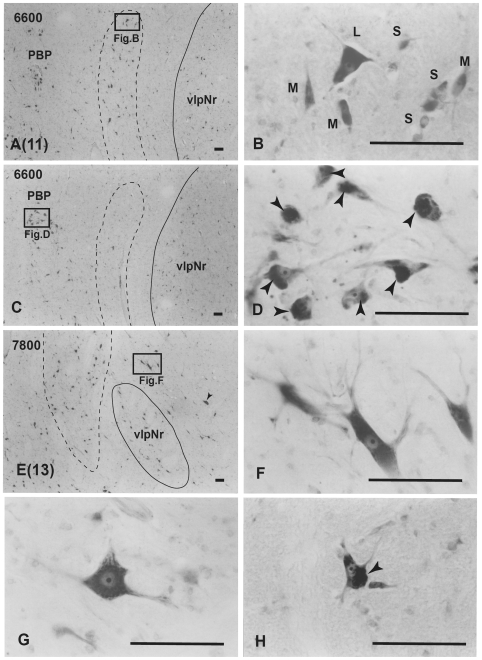
Human magnocellular red nucleus. Photomicrographs showing the distribution of Nissl-stained mNr cells. A (11) and E (13) correspond to sections 11 and 13 in Fig. 10. C is a reversed photomicrograph of the contralateral side of A for comparison with C. The area indicated by a broken line is the outer layer of the semi-lunar shell of mNr (A, C and E). It exits between the capsule of the superior cerebellar peduncle and the parabrachial pigmented nucleus (PBP). B. High magnification views of large (L), medium (M) and small (S) neurons of the outer shell of mNr. D. Many pigmented cells exist in the parabrachial pigmented nucleus. Arrowheads indicate accumulation of pigment. E. Caudal end of vlpNr. Arrowhead indicates one giant neuron. F and G. Giant neurons among fibers of the superior cerebellar pedunculus. H. Large neuron contains pigments. Arrowhead indicates accumulation of pigment. The number in the left corner of each photomicrograph is the rostrocaudal distance (in micrometers) from the rostral tip of the red nucleus. PBP–parabrachial pigmented nucleus, vlpNr–ventorlateral part of parvicellular red nucleus. Scale bars = 100 µm in A–H.

Using a series of sections prepared with a myelin (Figs. 10 and 11D), and Nissl (Figs. 10, 11 and 12) stain, we observed the mesodiencephalic nuclei consisting of the ND and the red nucleus. Myelin staining of brain sections shows that the fiber bundles of the superior cerebellar peduncle penetrate the center of the red nucleus at an angle from a caudo-medial to rostro-lateral direction and also wrap around the outer surface of the red nucleus like a capsule (Fig. 10). The FR penetrates and contacts the dorsomedial part of the red nucleus (sections 2–4 in Fig. 10). This dorsomedial part of the red nucleus is considered as corresponding to the human NB because this oval area shows microscopical-level features (i.e., more densely packed cells) that correspond more to the periaquaductal gray matter than they do to the reticular formation, similar to what is seen in the feline NB (see also Fig. 11D). Furthermore, fine myelinated fibers vertically rose from the NB and joined the medial part of the CTT (Fig. 11D). We conclude that the FR takes a radically different course with respect to the NB compared to cat and macaque: i.e., in the human the FR passes through the medial side of the NB, whereas in the cat and macaque, the FR passes through the lateral side of the NB and dmpNr. At this level, the ND is separated from the red nucleus by fiber bundles consisting of the superior cerebellar peduncle, CTT, MTT and MLF and is thus isolated in the ventral central gray (Figs. 10B–D, sections 4–8 in Fig. 10).

At the level of the oculomotor nucleus, oculomotor nerve fibers run along the surface of the red nucleus whereas caudally the many fiber bundles of the superior cerebellar peduncle penetrate the tail of the red nucleus (Figs. 10E–G, sections 10–14 in Fig. 10). Some rubral neurons remain at the ventrolateral part (Fig. 10G, section 12 in Fig. 10) of the tail of the red nucleus; this corresponds to the caudal part (section N in Fig. 2) of the cat's vlpNr. Efferent fibers from rubral neurons emerge from the dorsomedial part of the red nucleus, penetrate the cellular layer and its outer capsule of the fiber bundle, and then descend as the CTT to the inferior olive. The medial part of this CTT contains fibers emerging from the NB (Fig. 11D). Giant as well as large neurons of the mNr are gathered largely into a dorsal and a ventral group and are scattered among the fibers of the superior cerebellar peduncle (see section 14 in Fig. 10, Fig. 12). These neurons contain coarse Nissl bodies (Fig. 12F and G).

At the caudal level of the vlpNr, large, medium and small sized neurons were seen to be gathered between the outer capsule of vlpNr and the pigmented (see next paragraph) cell layer (Fig. 12A–E). This neural zone and scattered giant and large neurons of the mNr form the semi-lunar shell which might correspond to the well-developed semi-lunar shell of mNr seen in infants (Ulfing and Chan, 2001; 2002 [12], [13]; Yamaguchi and Goto, 2006 [14]). This adult mNr zone was vestigial, since in some areas these neurons had almost disappeared (Fig. 12C).

Many large, pigmented neurons are observed in the surrounding area outside the red nucleus and superior cerebellar peduncle, such as the parabrachial pigmental nucleus and the rostral and caudal linear nuclei (Fig. 12C and D). There is the possibility that some large pigmented neurons near the red nucleus are mis-identified as mNr neurons. However, these neurons can be excluded clearly from being mNr neurons since cell bodies of the former contain neuromelanin pigment while the latter do not (Fig. 12F, G and H).

## Discussion

### Neural Sheet Model of the Parvicellular Red Nucleus (Fig. 13)

Ogawa (1939) attributed the origin of the MTT in the cat to three structures: the ND, Nint, and nucleus of the fields of Forel (corresponding to the rostral interstitial nucleus of MLF) and concluded that the human red nucleus corresponds to these nuclei [1]. Thereafter, many researchers subscribed to the view that quadrupedal animals have a well-developed MTT and that humans have a well-developed CTT; they furthermore held that both tracts have the same origins and terminations (see reference in Voogd, 2003 [15]). Cells of origin of these olivary projections have been identified with the HRP method. The subparafascicular and parafascicular nuclei in the cat (as defined by [16], [17], [18], [19], [20], [21], [22]) have been redefined as the rostral part of the ND, a structure that projects to the rostral MAO (Onodera, 1984 [3]). The rostral part of the ND in the rat (as shown by Ruigrok and Cella, 1995 [23]) also projects to the rostral MAO (Onodera et al., 2004 [24]). However the rat's ND proper does not project to the inferior olive [23], [25] and does not receive terminals from the substantia nigra pars reticulata (SNr) which projects to the ventrolateral central gray ventral to the rat's ND proper (Gerfen et al., 1982 [26]; Deniau and Chevalier, 1992 [27]) in contrast to the cat's ND which projects to the rostral MAO (Onodera, 1984 [3]) and receives terminals from the SNr (Onodera and Hicks, 1998 [28]). Therefore, there is no neuroanatomical homology between the rat's ND (proper) and the feline ND. As the rat's ventrolateral central gray, ventral to the ND proper, receives terminals from the SNr [27], [26] and projects to the inferior olive [23], [25], we consider that this region of the ventral central gray corresponds not to the cat's NB but the cat's ND.

**Figure 13:**
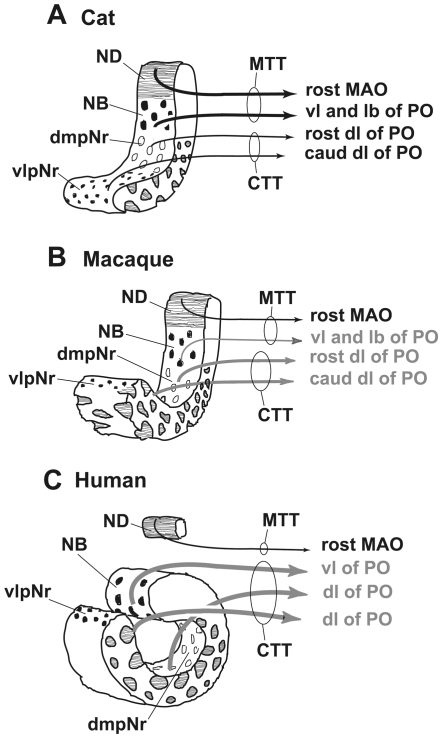
Neural sheet model of red nucleus. A. In the cat, the poorly-developed neural sheet curves. B. In the macaque, the moderately developed neural sheet curves. C. In the human, the extremely well-developed neural sheet is completely rolled. The human NB is defined as the dorsomedial part of the red nucleus and it projects to the vl of PO via the medial part of the CTT. A strong stream of these well-developed CTT fibers separates the NB from ND. The gray color indicates a “still uncertain” projection. CTT–central tegmental tract, dmpNr–dorsomedial part of parvicellular red nucleus, lb–lateral bend, MAO–medial accessory olive, MTT–medial tegmental tract, NB–nucleus accessorius medialis of Bechterew, ND–nucleus of Darkschewitsch, PO–principal olive, vl–ventral lamella, vlpNr–ventorlateral part of parvicellular red nucleus.

In an autoradiographical study, Saint-Cyr and Courville (1982) [19] and Holstege and Tan (1988) [29] were not able to define a detailed topographic organization within the mesodiencephalo-olivary projections in the cat. By contrast, our previous study in the cat presented findings that showed a precise topographical organization within these olivary projections and a well-developed MTT belonging to the well-developed ND and NB and a poorly-developed CTT belonging to the poorly-developed dm- and vl-pNr (Onodera, 1984 [3], see Fig. 13A). We then confirmed that in cats, the ND projects by way of the MTT, while the Nint and the rostral interstitial nucleus of MLF project only to the MLF (Onodera and Hicks, 1998 [28]). Our data from this study in the cat show that the border among ND, NB and dmpNr has become clear and that this well-restricted NB area strongly projects to the lateral bend of the PO more than to the vl of PO, judging from a comparison of case 1 with case 2.

A question of interest at this point was whether quadrupeds other than the cat also have a similar topographically organized olivary projection from their mesodiencephalic regions. Previous studies in the rat (Swenson and Castro, 1983 [30]), macaque (Strominger et al., 1979 [31]) and chimpanzee (Strominger et al., 1985 [32]) suggested that they do. As the so-called “rat's rostral ND” projects only to the rostral MAO in the rat, as supported by use of the DiI method (Onodera et al., 2004 [24]), we propose that the rat also has a similar topographically precise projection of the mesodiencephalo-olivary system. The macaque, a species that also practises quadrupedal locomotion but has a manipulative hand, possesses a well-developed pNr and a well-developed mNr. Using autoradiographic tracing methods, Strominger et al. (1979) have demonstrated that the lateral and medial parts of the macaque's pNr projects to the dorsal lamella (dl-) and ventral lamella of (vl-) PO, respectively, and the intermediate part projects to the bend region of PO [31]. In that study medial and intermediate parts of the pNr contain the NB. Our present data from the macaque show that 1) the dmpNr projects to the rostral part of dl of PO in case 3; 2) the vlpNr projects to the caudal part of dl of PO in case 4; and 3) the rostral ND projects to the caudal part of the rostral half of MAO in case 4. Our previous study in the cat (Onodera, 1984 [3]) showed the same topographical pattern of the projection from the subnuclei of pNr to the dl of PO. However, the possibility that dmpNr and vlpNr project to vl- and dl of PO, respectively still remains. We also showed the point to point projection between the ND and the rostral half of MAO: i.e., in the cat, the medial part of the ND projects to the lateral part of the rostral MAO and the lateral part of the ND to the medial part of the rostral MAO (Onodera and Hicks, 1995 [33]). The present data from the macaque show a new rostro-caudal relationship between the ND and rostral MAO. In macaque, the rostral and caudal part of the ND project to the caudal and rostral part of the rostral MAO, respectively. Therefore the topographical relationship of projections connecting the ND and the rostral MAO is inversely related. Based on these considerations it appears reasonable to propose that the mesodiencephalo-olivary projections of cat and macaque have essentially the same pattern, although there is a difference in the pNr of the cat. Therefore we conclude that fundamentally, quadrupeds have a similar topographical olivary projection from the mesodiencephalic nuclei as we know is the case for the cat (compare Fig. 13A and B).

By contrast, the human red nucleus as well as that of the chimpanzee shows other specialized features, i.e., an anatomically isolated ND alongside a well-developed, egg-shaped red nucleus. Comparative neuroanatomical studies of primates have shown that the separation of ND and NB began at the divergence point between ape and monkey [34]. The marmoset and macaque possess a continuous zone consisting of the ND, NB and pNr, similar to the cat. The gibbon represents a transition species with respect to the connection between ND and its prominently well-developed NB. Such a well-developed NB and dmpNr may have been related to the evolutionary development of the frontal cortex containing the FEF, since the NB and its adjacent dmpNr receives a projection from the FEF and its adjacent frontal cortex in macaque [35], [36], [37], [5], [38]. As with the human, the chimpanzee exhibits a complete separation between ND and its well-developed red nucleus [34].

Actually the human red nucleus does not present the same appearance as the neural sheet-like appearance of the cat's and monkeys red nucleus. However the human red nucleus likely has fundamentally the similar topographical relationship as that of the cat, judging from the comparison between human brain materials (Nathan and Smith, 1982 [39]; Voogd, 2003 [15]) and experimental data, especially those from the chimpanzee's brain (Strominger et al., 1985 [32]). The latter have shown that the chimpanzee's CTT projects only to the PO. The present study with myelin stain has shown that the fine myelinated fibers vertically arise from the NB and join the medial part of the CTT (see Fig. 11D). This stands in contrast to the situation in the cat, where the NB occupies an area within the MTT (Onodera, 1984 [3]). Therefore the position of the human NB can be seen to have shifted from the ventral central gray into the reticular formation (Fig. 13C). Accordingly the zonal sheet of the human's red nucleus might have comprised longitudinally-organized components of a well-developed vl- and dm-pNr, and an extremely well-developed NB.

### Three-Dimensional Model of the Cat's and Macaque's Red Nucleus (Fig. 14A and B)

**Figure 14:**
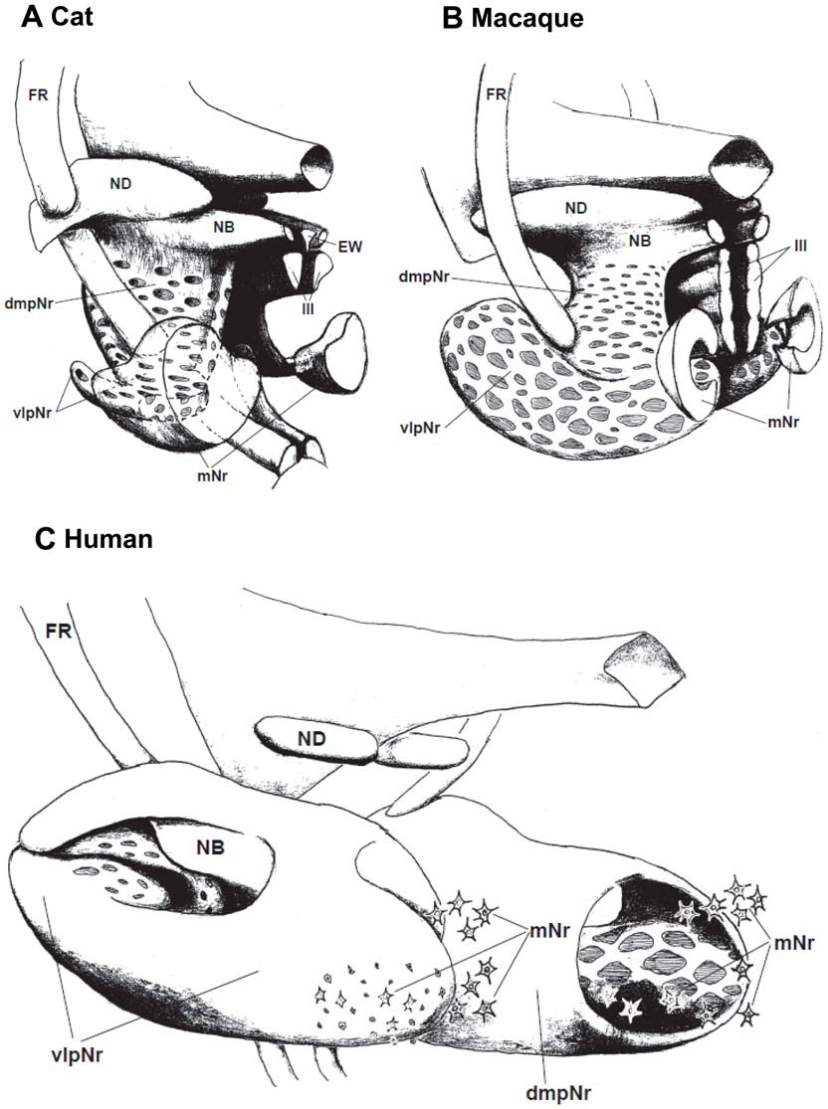
Three-dimensional models of red nucleus. The mesodiencephalic nuclei of the cat (A), the macaque (B), and the human (C) as reconstructed using serial sections shown in Fig. 1 for cat; Figs. 7,8 and 11 for macaque; and a myelo- and cytoarchitectonic analysis in Figs. 10–12 for human. The rostral ends of these modeles toward left side. dmpNr–dorsomedial part of parvicellular red nucleus, EW–Edinger-Westphal nucleus, FR–fasciculus retroflexus, mNr–magnocellular red nucleus, NB–nucleus accessorius medialis of Bechterew, ND–nucleus of Darkschewitsch, vlpNr–ventorlateral part of parvicellular red nucleus, III–oculomotor nucleus.

Our three-dimensional (3D) models (Fig. 14A and B) of the feline and macaque's red nucleus were enabled through the use of WGA-HRP-labelled serial sections from the cat (Fig. 2) and the macaque (Figs. 5, 6 and 9). The fundamental structure of the macaque's 3D model was the same as the cat's 3D model. The macaque's vlpNr is well-developed compared with the cat's vlpNr. Larger fiber bundles of the superior cerebellar peduncle penetrated more caudo-lateral parts of the pNr, while smaller fiber bundles penetrated more rostro-medial parts of the pNr.

### Rolled-Sheet Model of the Human Red Nucleus (Fig. 14C)

The two models of the human red nucleus based on cytoarchitecture and referred to here have been proposed earlier. The first model was introduced by Grofová and Maršala (1960) [40] and it represents a polyhedron shape that is covered by several irregular zones that have relatively greater cellular density. In the wide, central area of pNr there are rarely-seen scattered neurons that are separated by strong bundles of myelinated fibers. It is impossible always to identify unambiguously the form and extent of the above-mentioned zones in each case, as the criteria useful for the identification of each cellular zone are imprecise. It is useful to keep uppermost in mind that the human red nucleus is covered by a dense cellular layer.

The second model was published by Olszewski and Baxter (1982) [41] and it was described as consisting of three subnuclei: pars oralis, pars dorsomedialis and pars caudalis. The pars oralis is the oral pole of the red nucleus. The pars dorsomedialis is separated from oralis by a lamella of myelinated fibers and the cell density of these two parts is higher than in pars caudalis. The pars caudalis is traversed by many fiber bundles within which its constituent cells are loosely and irregularly arranged.

We would like to propose a third model we name as “rolled sheet”. This is based in part on a combined myelo- and cytoarchitectonic analysis, considered together with the above two preceding models, plus a consideration of the common topographical olivary projection (see Fig. 13). A dense cellular layer of the pNr envelops huge myelinated fiber bundles of the superior cerebellar peduncle. These fiber bundles branch off collateral terminals to the pNr neurons and pass through the center of the red nucleus in a dorsolateral direction. These passing fibers ascend through the dorsolateral opening to the thalamus and descend from the cortex. The caudomedial end of the red nucleus forms a ring-like structure allowing for the entrance of the cerebellar peduncle and the elongated bulge of the ventolateral wall (i.e., caudal end of vlpNr). The dorsomedial part of the rostral red nucleus is a densely packed cell mass that is divided by a lamella of myelinated fibers (see Fig. 11D). We defined this dorsomedial region and its caudal continuation as the NB. The rostral pole of the red nucleus forms a cell mass appearing like a set of lips (see Fig. 11A); dense cellular layers contact each other to look like closed lips and these possess a few penetrating myelinated fiber bundles. The outer surface of the red nucleus is formed by densely packed cells and furthermore is covered by a dense, myelinated fiber capsule; the inner surface of the rolled sheet is formed by loosely and irregularly arranged cells and is penetrated by large collateral fiber bundles of the superior cerebellar peduncle. Therefore the human red nucleus may be considered to be classified as a typical “closed nucleus” just as the cat's red nucleus is (Mannen, 1988 [2]), although there are other differences between the parvicellular red nucleus for the human and the magnocellular red nucleus for the cat.

Both giant and large mNr neurons are located in dorsal and ventral groups caudal to the vlpNr (see Figs. 10 and 12E, F and G). Some large, medium and small mNr neurons also are scattered outside the outer capsule of the red nucleus (Fig. 12A, B and E). These mNr neurons form a semi-lunar shell at the caudal end of vlpNr, and are considered a homologue of the cat's and macaque's mNr, judging from their positions (compare Fig. 14A, B and C). In the monkey, neurons with a coarse Nissl pattern (i.e., mNr neurons) extend for a short distance along the lateral side of fine neurons (i.e., pNr neurons) in the rostral part of the red nucleus [42]. This short dorsolateral extension of the monkey's mNr corresponds to the semilunar shell. The gibbon mNr is laminated and rotated about its major axis [43]. This feature seems equivalent to the semilunar shell. The chimpanzee mNr shows a similar feature as the human semilunar shell: i.e., populations of coarse and fine neurons occupying distinct and separate areas within a small junctional zone [44]. Recent studies concerning the human fetal mNr showed that this structure develops progressively during the latter half of gestation and that the mNr is more prominent in fetal stages than in adulthood (Ulfing and Chan, 2001; 2002 [12], [13]; Yamaguchi and Goto, 2006 [14]). The fetal mNr drapes around the caudal third of the pNr in the form of a semi-lunar shell, and it and the pNr are clearly separated from each other.

Pompeiano and Brodal (1957) pointed out the possibility of underestimates having been made of the extent of the human rubrospinal projection, if a substantial number of rubrospinal neurons were small [45]. Papez and Stottler (1940) showed that in human infants, small cells located caudal and lateral in the red nucleus project to the contralateral spinal cord [46]. Using tissue from an adult patient with an old paramedian infarct involving the red nuclei, Terao and co-workers (1996) reported that small myelinated fibers in the lateral corticospinal tract were selectively diminished at all spinal cord levels compared with large myelinated fibers. They suggested that some of these diminished small myelinated fibers may correspond to the human rubrospinal tract [47].

The human red nucleus was previously considered to correspond to the rubral complex of nuclei. In quadrupeds, this complex consists of the mNr and the mesodiencephalic nuclei projecting to the inferior olive via both the CTT and MTT (for rat, see Ruigrok and Cella, 1995 [23]; for cat, see Ogawa, 1939 [1]). However the present study shows clearly that the CTT components (i.e., NB-pNr complex) and the rudimentary semi-lunar shell of mNr actually correspond to the human red nucleus.

### Evolutionary Consideration

Although it is believed generally that the primate mNr shows a consistent trend toward gradual functional and anatomical diminution during the progress of evolution, as a matter of fact this is not the case. Those primates that are proficient at terrestrial quadrupedalism exhibit a very well-developed mNr. Examples are the macaque and baboon [42], [43]. Both corticorubral and corticospinal neurons mix well in the motor cortex of such monkeys. However, the mNr of apes (e.g., gibbon and chimpanzee) shows a consistent trend to decrease gradually along the phylogenetic scale [48], [49], [43], [50]. Correspondingly, the corticorubral neurons of these apes may have followed a gradual decrease. This reduction of the role of the mNr is considered to be a consequence of a dynamic continuous change in cerebral organization that is furthered by neuronal competition. The chimpanzee's mNr is the smallest and most limited in size for quadrupedalism and arboreal life. Anatomical data (Sobel 1977 [44]) have shown that the cell count in the chimpanzee's mNr (ca. 1,250) is 2.8 times greater than that in humans (ca. 450 cells). Neurons of the human mNr are found in a variety of sizes: giant, large, medium and small cells [44]. Humans are known to possess 150–200 giant-to-large sized neurons in that portion of the mNr which project large myelinated fibers not only to the brain stem but also within the first three cervical segments (Nathan & Smith 1982 [39]). Therefore there must remain the possibility that there were these mNr neurons related to limb control associated with propriospinal neurons in upper cervical segments, and that these projected extensively throughout the cervical enlargement (Nathan et al. 1996 [51]), being related to shoulder movement [37].

Our previous studies [3], [33], [28], [52], [24] have shown that the mesodiencephalic nuclei, including the ND, NB and pNr, show developmental differences corresponding to species-specialized body parts, such as the human hand, the elephant's trunk and the whale's axial musculature system. Onodera and Hicks (1999) [52] proposed that the phylogenetically newer pyramidal tract and well-developed mesodiencephalo-olivo-cerebellar circuits “broke through” the constraints of the older rubrospinal system of the mNr and suggested that the human pyramidal system took over to provide a tonically active framework for locomotor function, rather than actually physically replacing the rubrospinal system.

The transient prominence of the fetal and infant mNr discussed above might have provided the organism with a functional rubrospinal projection. This may have permitted the transition from the rubrospinal system to corticospinal system: i.e., subsequent normal development of the corticospinal system during ontogeny. It is known from current observation of human infants that once certain key milestones are achieved for stabilization of each body segment (the head, upper and lower torso segments and crawling behavior), standing is achieved, balancing behavior performed, as is low-velocity, bipedal locomotion that typically begins around one year of age. The acquisition of bipedalism demands not only the maturation of the body's phenotype but also the reorganization of the nervous system. Clearly evolution cannot occur by transformation from one adult form to another adult form, but by ontogenetic change over time. Humans have taken several million years to establish bipedalism through changes of the genetic pool, while individual humans after birth take several years to establish the same process, of course without any genetic change.

**Figure 15:**
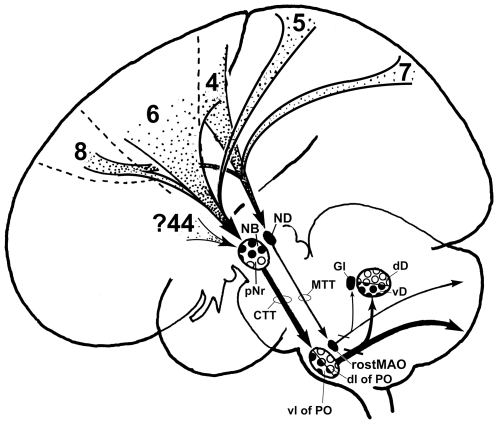
Human cerebro-cerebellar system. The medial cerebellar system receives cortical information from areas 4, 8 and 7 via the ND [62], [63], [38], [56]. The lateral cerebellar system might not have been related only to skilful motor learning via areas 4, 6, 5 and 8 [35], [36], [63], [38], [56], but also to cognitive and language processing via the prefrontal cortico-rubral projection. CTT–central tegmental tract, dD–dorsal half of cerebellar dentate nucleus, dl–dorsal lamella, Gl–cerebellar globose nucleus, lb–lateral bend, MAO–medial accessory olive, MTT–medial tegmental tract, NB–nucleus accessorius medialis of Bechterew, ND–nucleus of Darkschewitsch, pNr–parvicellular red nucleus, PO–principal olive, vD–ventral half of cerebellar dentate nucleus, vl–ventral lamella, 4–motor area, 5–somatosensory association cortex, 6–premotor area, 7–posterior parietal cortex, 8–frontal eye field, 44–Broca's speech area.

Using voxel-based morphometric analyses, Vargha-Khaden and her coworkers (2005) showed that the affected KE family members with a higher-order orofacial motor impairment during speech have significantly reduced grey matter in the inferior frontal gyrus (Broca's area), the precentral gyrus, the temporal pole, the head of the caudate nucleus and the ventral cerebellum (lobules VIIB and VIIIB) [53]. In the normal human fetus, the *FOXP2* gene is expressed in not only the cerebral cortex, basal ganglia and thalamus, but also the cerebellum, inferior olivary complex and red nucleus (Lai et al., 2003 [54]; Teramitsu et al., 2004 [55]). These data propose that one circuit for *FOXP2*-dependent speech and language might be not only the prefronto-caudate-loop but also the inferior fronto (Broca's area)- rubro-olivo-lateral cerebellar loop. The NB may receive projections from ventral area 6, within and caudal to the inferior ramus of the arcuate sulcus (see Leichnetz et al, 1984 [56]). As this region (area F5) corresponds to human area 44 (the inferior frontal cortex) as judged through comparative architectonic analysis (Petrides and Pandya, 1994 [57]), the NB might be related to Broca's area, so it is not only the function but also the position of NB that changes between macaque and human. The present study suggests that the well-developed human NB might be related to language via its connections with Broca's area 44 (the inferior frontal cortex) and the well-developed ventral half of the cerebellar dentate nucleus (see Fig. 15), the latter which is developed more greatly than the ape's ventral half of the cerebellar dentate nucleus (see Matano, 2001 [58]). A recent diffusion tensor tractography study using MRI demonstrated that the human red nucleus receives strong projections from the prefrontal cortex (Habas and Cabanis, 2006 [59]).

### Conclusions

The present study has employed the cat and macaque to show that: 1) the topographical relationship between the ND and rostral MAO is inversely related: i.e., the rostral and caudal parts of the ND project to the caudal and rostral parts of the rostral MAO respectively, while the medial and lateral parts of the ND project to the lateral and medial parts of the rostral MAO, respectively; 2) the NB projects strongly to the lateral bend of the PO: 3) the dmpNr and vlpNr project to the rostral and caudal part of dl of PO, respectively. However the possibility that macaque's dmpNr and vlpNr project to vl and dl of PO, respectively still remains.

Our treatment and analysis of human material showed that the human NB may be separated from the ND in the ventral central gray, and phylogenetically may have been translocated into the present roll-shaped red nucleus in the reticular formation.

Further study of the pNr and NB of experimental animals will demonstrate more precise topographical olivary projections and may facilitate development of a very useful model for comparison with research on the human red nucleus. This would allow us to break through the limitations necessarily imposed upon future neuroanatomical and physiological investigations of the human red nucleus.
